# Export Expansion May Increase Adult Illness and Injury: A Quasi-Natural Experiment on China's Accession to the World Trade Organization

**DOI:** 10.3389/fpubh.2022.798686

**Published:** 2022-04-05

**Authors:** Hongwen Chen, Junbing Xu, Jianzheng Liu

**Affiliations:** ^1^Department of International Economics and Business, School of Economics, Xiamen University, Xiamen, China; ^2^New Huadu Business School, Minjiang University, Fuzhou, China; ^3^School of Public Affairs, Xiamen University, Xiamen, China

**Keywords:** export shock, WTO, China, difference-in-differences, CHNS, adult illness/injury

## Abstract

**Purpose:**

Exports can boost the economy, but may also cause harm to health through, for example, increased pollution and working hours. Although academic research extensively covers the impact of trade on health, few studies examine the mechanisms through which export expansion affects adult illness or injury within the past 4 weeks (illness/injury).

**Method:**

We utilized China's entry into the World Trade Organization (WTO) as a quasi-natural experiment to investigate the relationship between export expansion and adult illness/injury. We explored the possible mechanisms and the heterogeneity of these associations. Our methodology was based on the analysis of China's Health and Nutrition Survey data, Chinese Customs databases, and China's Statistical Yearbook.

**Results:**

Export expansion, induced by China's accession to the WTO, has a significantly positive effect on adult illness/injury [average effect (AE): 1.83%; 95% CI: 0.38–3.28%]. Our results remain robust following a series of robustness tests. Moreover, the effects of export expansion on adult illness/injury are more pronounced among urban residents (AE: 5.32%; 95% CI: 2.46–8.18%), women (AE: 2.68%; 95% CI: 0.57–4.80%), and higher-income groups (AE: 5.90%; 95% CI: 2.53–9.27%).

**Conclusions:**

We find a statistically significant and positive effect of export expansion on adult illness/injury.

## Introduction

Export-oriented policies are standard economic stimulus policies for countries with late-developing economies. Exports promote economic growth and play an important role in economic development, but they also introduced environmental and health problems. Although an extensive literature has examined the impact of trade on health (1–7), the other ways in which the export trade may affect adult illness/injury are not fully understood, especially in the context of developing countries with fast-growing export trade, such as China. China experienced a sharp increase in export volume after accession into the World Trade Organization (WTO). However, the extant literature on China's accession to WTO mainly focused on firm production, labor markets, or trading partners (8–16), and few studies examine the effect of export expansion on the illness/injury of Chinese adults. Thus, the purpose of this research is to examine the relationship between export expansion and adult illness/injury and explore why and how to export expansion resulting from trade liberalization affects adult illness/injury.

We focus on China because, on one hand, it is the world's most populous developing country and simultaneously, the largest exporter, making it crucial to explore the impact of trade policy on adult illness/injury. On the other hand, WTO accession provides an excellent opportunity to study Chinese adults' illness/injury fluctuations stemming from export expansion, as national-level policies, such as WTO accession, are relatively exogenous to individual illness/injury. Moreover, when China joined the WTO in late 2001, export shocks varied across regions. Therefore, we can use this regional variation to explore the impact of export expansion on adult illness/injury. Specifically, we empirically analyze the aforementioned effect with a difference-in-differences (DID) model. The main finding is that export expansion had a significantly positive effect on adult illness/injury, a result that remains robust after several tests, such as redefining the study sample by limiting the age range, changing the econometric model, reallocating experimental and control groups, adjusting the clustering standard errors, conducting parallel trend tests, and controlling for other simultaneous policies.

Additionally, this article explores export expansion's effects on adult illness/injury from the perspectives of pollutant emissions and the labor market. We find that industrial sulfur dioxide emissions and labor hours are the mechanisms through which export expansion associated with WTO accession increases adult illness/injury. Furthermore, the heterogeneity analysis shows that the effects of export expansion on adult illness/injury are more pronounced among urban residents, women, and higher-income groups.

This study contributes to several strands of literature. First, several scholars have examined the relationship between trade and economic activity (17–21), while others are investigating the interaction between trade and health. This study adds to the research examining trade liberalization's effects on adult health in terms of import competition (22–25), export expansion (26), and tariff shocks on intermediate goods (27). Specifically, several studies find that import competition has negative effects on workers' mental, physical, and general health (22–25). Hummels et al. (26) use worker-firm matching data from Denmark to explore the impact of export growth on workers. They reveal that export expansion reduced the number of sick days, and increased the number of working hours, injury rates, and sickness rates, particularly for female workers. However, they did not discuss the mechanisms. Additionally, most of these studies focus on developed countries, with a few exceptions, such as Fan et al. (27), who examine the impact of China's declining import tariffs on employee health. Thus, we contribute to this strand of literature by studying the effect of China's export expansion on adult illness/injury and discussing, in detail, the mechanisms behind this effect from the perspectives of pollutant emissions and the labor market.

Second, our article adapts to the research on how globalization affects children's health. The existing literature mainly examines this relationship in terms of income (28), environmental degradation (29), and the time spent on caregiving (30). For instance, Levine and Rothman (28) discuss the impact of trade on child health in terms of income and based on country-level data, and find that trade openness slightly reduced child mortality. Using export shocks and pollution export shocks, Bombardini and Li (29) study the effect of export expansion on infant mortality, based on the prefecture-level city data in China. They reveal that pollution export shocks significantly increased infant and child mortality, but export shocks helped reduce mortality rates, although this result was not robust. Furthermore, Dai et al. (30) use the China Health and Nutrition Survey (CHNS) data to explore export expansion's effect on the health of Chinese children under 6 years of age, and they find significant increases in child illness rates. We take these aforementioned elements into consideration and adapt them to the study of adult illness/injury. Accordingly, our article demonstrates that globalization might affect adult illness/injury through different channels.

Third, our work contributes to the literature examining trade's effect on human health in the field of environmental science (1–3). For example, Jiang et al. (1) find that export trade caused 12% of PM_2.5_-related deaths in China in 2007, Wang et al. (2) specify that trade caused 208,500 deaths in China in 2007, due to increased PM_2.5_ concentrations, and Ou et al. (3) discover that ozone pollution from Chinese exports caused 16,889 deaths in 2013. The abovementioned literature uses atmospheric chemical transport models to simulate changes in air pollutant concentrations stemming from trade and epidemiological models to calculate the deaths related to air pollutants. In contrast, we use econometric models to identify a causal relationship between export expansion and adult illness/injury.

Fourth, our study contributes to the literature examining the health impacts of trade policies in the field of public health (4–7). For instance, Olper et al. (5) use the data from emerging and developing countries to examine the impact of trade liberalization on child mortality. They determine that trade liberalization reduced child mortality on average significantly. Barlow et al. (6) use the synthetic control method to analyze the impact of trade liberalization on child mortality in 36 low- and middle-income countries from 1963 to 2005, and find that trade liberalization can create opportunities to reduce child mortality. Our study makes a contribution through an examination of export-oriented policies specifically whilst a few existing articles have examined this specific subset of trade policies.

The remainder of this article is structured as follows. Policy Background provides the policy background on trade liberalization, and Empirical Strategy and Data describes the data sources and empirical strategy. Results presents the benchmark results, as well as robustness, mechanism, and heterogeneity analyses. Finally, Discussion provides conclusions and implications.

## Policy Background

**Figure 1:**
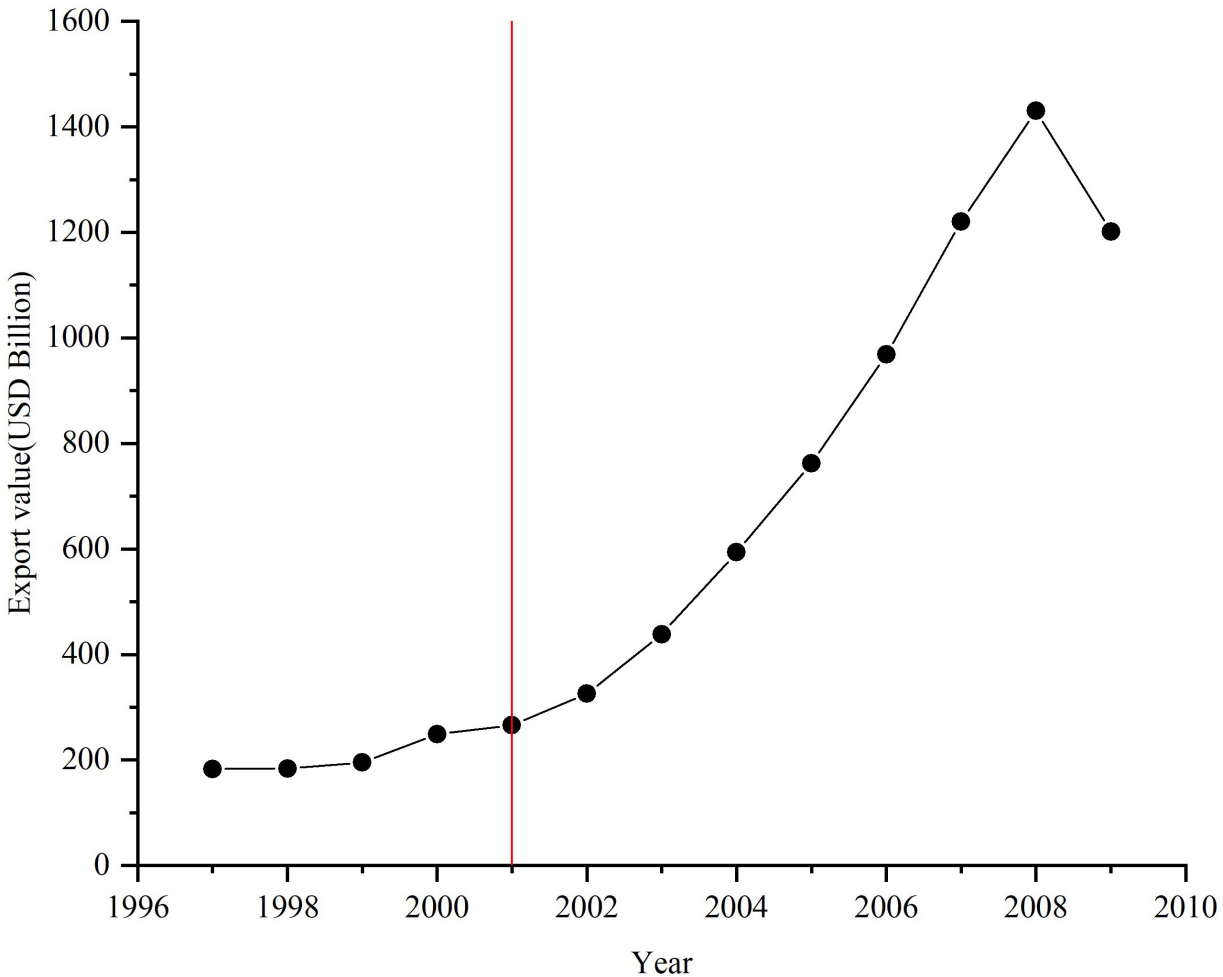
Export value in China between 1997 and 2009.

In 1986, China applied for reinstatement as a contracting party to the General Agreement on Tariffs and Trade, and in July 1995, it formally applied to the WTO for accession. When China became a member of the WTO in December 2001, it had already taken several measures to open up to the rest of the world, including encouraging domestic companies to export and lowering tariffs. In other words, China began to reduce tariffs well before it joined the WTO, from 42.9% in 1992 to 17% in 1997 (27), and between 1997 and 2001, the tariffs fell only marginally. This series of trade promotion measures led to a dramatic increase in China's export trade after its accession to the WTO. Figure 1 illustrates a significant increase in China's export value from 1997 (US$182.79 billion) to 2009 (US$1201.61 billion). Although China's export value growth from 1997 to 2001 was not significant, the value rose significantly from 2002 to 2008 (339.41%), decreasing slightly from 2008 to 2009, due to the financial crisis. As China is a vast country, economic development levels and cultural customs vary significantly from region to region. Thus, while China's overall export value may rise, its growth rate can vary per region.

## Empirical Strategy and Data

### Empirical Strategy

In this article, we examine whether an individual's probability of experiencing an illness or injury in the past 4 weeks is associated with living in a region undergoing export expansion, using a region's export/gross domestic product (GDP) ratio as a proxy. The dependent variable, *Illness/injury*, represents an individual's illness/injury condition. The adult illness/injury is assessed from the CHNS database, which indicates whether a person has been ill or injured in the last 4 weeks. If a person is sick, then the indicator is 1, otherwise 0. CHNS conducts a survey only once every year in which respondents are asked, “Have you been ill or injured in the last 4 weeks?”. Therefore, the variable of illness/injury is annual data, indicating the illness/injury status of the respondents in the previous month. Because the 4 weeks were randomly selected, it is possible to assess the illness/injury status of the respondents during a certain period of time, which is representative of their illness/injury status this year. The database has been used in various studies to examine the health of the population (27, 30). *Export_Ratio* and *WTO* are dummy variables. When a region's export/GDP value in 2001 is higher than the median value, *Export_Ratio* equals 1, otherwise 0. *WTO* equals 1 for the years from 2002 afterward and 0 for the other years. The data on export are sourced from the Chinese Customs Databases, and the data on GDP are derived from the National Bureau of Statistics.

Following Bombardini and Li and Fan et al., we included four types of control variables from micro to macro (27, 29): (1) $X_{i}^{{}^{\prime }}$ denotes individual-level characteristics, such as age, education level, gender, height, weight, and social security participation; (2) $X_{h}^{{}^{\prime }}$ represents the household-level variable, including household access to running water, indoor or outdoor toilets, electricity, and household income; (3) $X_{c}^{{}^{\prime }}$ reflects the community-level variables, such as population density, health service quality, and sanitation scores; and (4) $X_{p}^{{}^{\prime }}$ denotes region-level differences, such as GDP per capita and the share of secondary industries. We obtained individual, household, and community-level data from the CHNS database and regional level data from the National Bureau of Statistics. Finally, λ_*c*_ reflects the community fixed effects, which controls for all time-invariant community characteristics. λ_*t*_ represents the year fixed effects capturing yearly shocks common to all individuals. ε_*ihcpt*_ is the error term, and the SD is clustered to the individual level to address potential heteroskedasticity (31).

**Figure 2:**
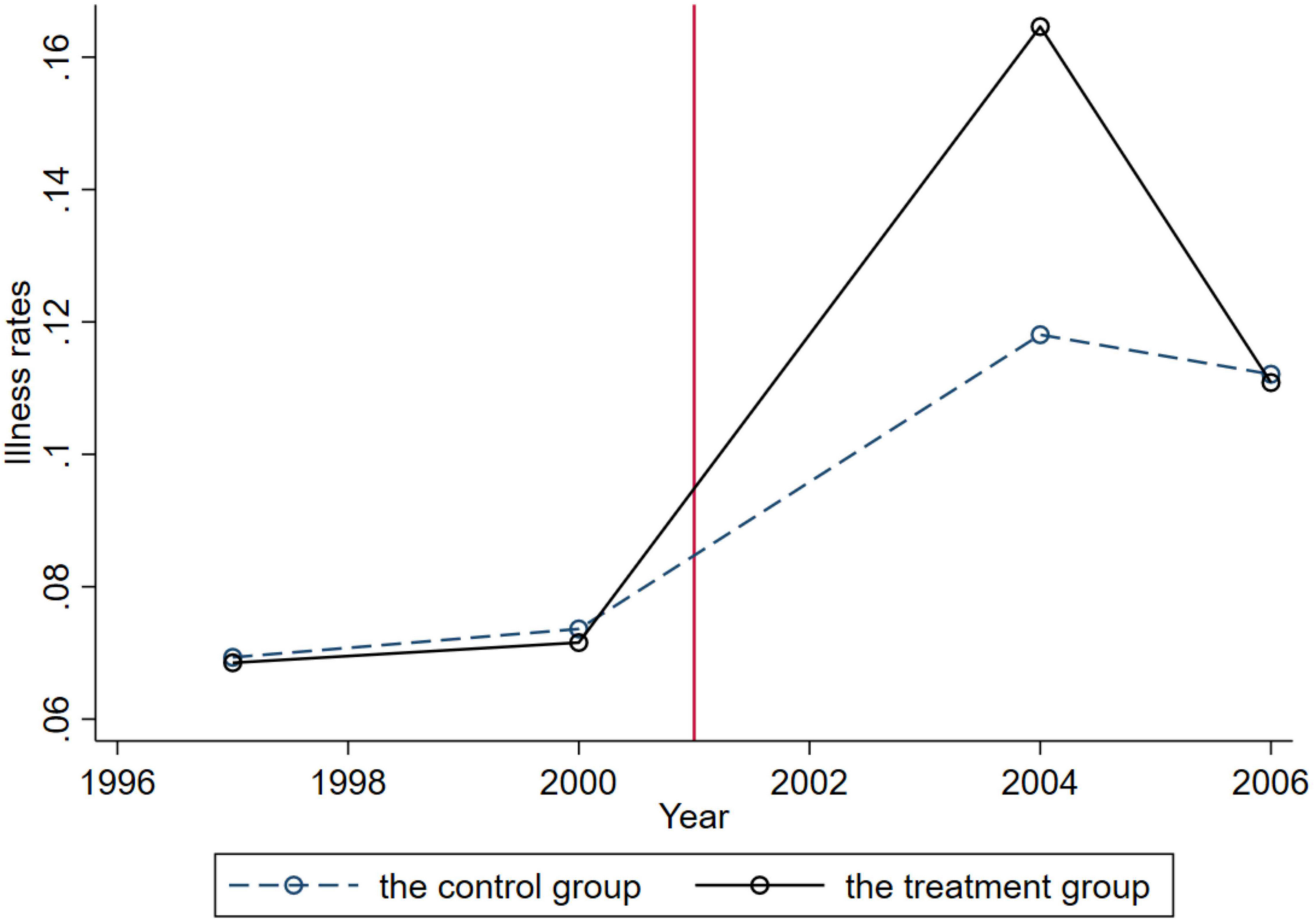
Illness rates in treatment and control groups by years, 1997–2006.

Based on the DID method, we investigate the impact of export shocks on adult illness/injury in the context of China's accession to the WTO in 2001. Here is a theoretical explanation of why we distinguish the treatment group and the control group based on the export/GDP ratio. We can measure the probability of ill [i.e., *p*(*h*)], which we calculate as the number who are ill (i.e., *nh*) divided by the total number (i.e., *nt*). Thus, we can obtain *p*(*h*) = *nh/nt*. We hypothesize that *nh* is a function of the level of exports, which implicitly we see as being the number who work in exports (i.e., *nx*) multiplied by the probability that an increase in exports worsens their health [i.e., *p*(*hx*)]. Therefore, we can get *nh* = nx^*^p(*hx*). Of course, *nh* depends on other things rather than exporting, but we can basically eliminate these from the exercise by using DID. Hence, we can get *p*(*h*) = nx^*^p(*hx*)*/nt* = sx^*^p(*hx*), where *sx* is the share of workers in exporting. We view the export/GDP ratio as a proxy for *sx*. Therefore, we use the export/GDP ratio to differentiate the experimental group from the control group. The DID method is able to deal with the endogenous issue resulting from mutual causation and omitted variables, improving the accuracy of identification. Based on the probit model, we devise the DID estimation equation as follows:


(1) $$\begin{array}{l} \text{Illness}/{\text{injury}}_{ihcpt}\;=\;\alpha +\beta Export\_Ratio_{p}\;\times WTO_{t}\\ \;+\;\theta Export\_Ratio_{p}+\rho WTO_{t}+X_{i}^{{}^{\prime }}\delta +X_{h}^{{}^{\prime }}\gamma \\ \;+X_{c}^{{}^{\prime }}k+X_{p}^{{}^{\prime }}\eta +\lambda_{c}+\lambda_{t}+\varepsilon_{ihcpt} \end{array}$$

where *i, h, c, p*, and *t* denote individual, household, community, province, and time, respectively. We expect β to be positive if export shocks caused by trade liberalization increased adult illness/injury. To examine whether the illness rates of treatment and control groups have the same trend before China's accession to the WTO, we plot time trends for the two groups' average illness rates in Figure 2. Both groups showed the same trend before the accession, but there is a clear divergence in their illness rate trend after the accession. Specifically, in the period 2000–2004, the illness rates of the treatment group increased at a much higher rate than that of the control group. There has been a significant decline in the average illness rates of the experimental group since 2004, which may reflect the fact that short-term export shocks are harmful, but long-term export shocks may be beneficial. This study focuses primarily on the short-term effects of sudden activities.

### Data

Our individual-level data are the panel data from the CHNS, a follow-up survey conducted by the Carolina Population Center at the University of North Carolina at Chapel Hill in collaboration with the National Institute for Nutrition and Health at the Chinese Center for Disease Control and Prevention. The CHNS was designed to examine the impact of health, nutrition, and family planning policies and programs implemented by national and local governments to understand how the social and economic transformations of Chinese society affect its population. Due to the outstanding quality of CHNS data, various studies use the database to investigate population health (27, 29–34). The survey started in 1989, with several subsequent rounds in 1991, 1993, 1997, 2000, 2004, 2006, 2009, 2011, and 2015. The years covered in this article are 1997, 2000, 2004, and 2006.

**Figure 3:**
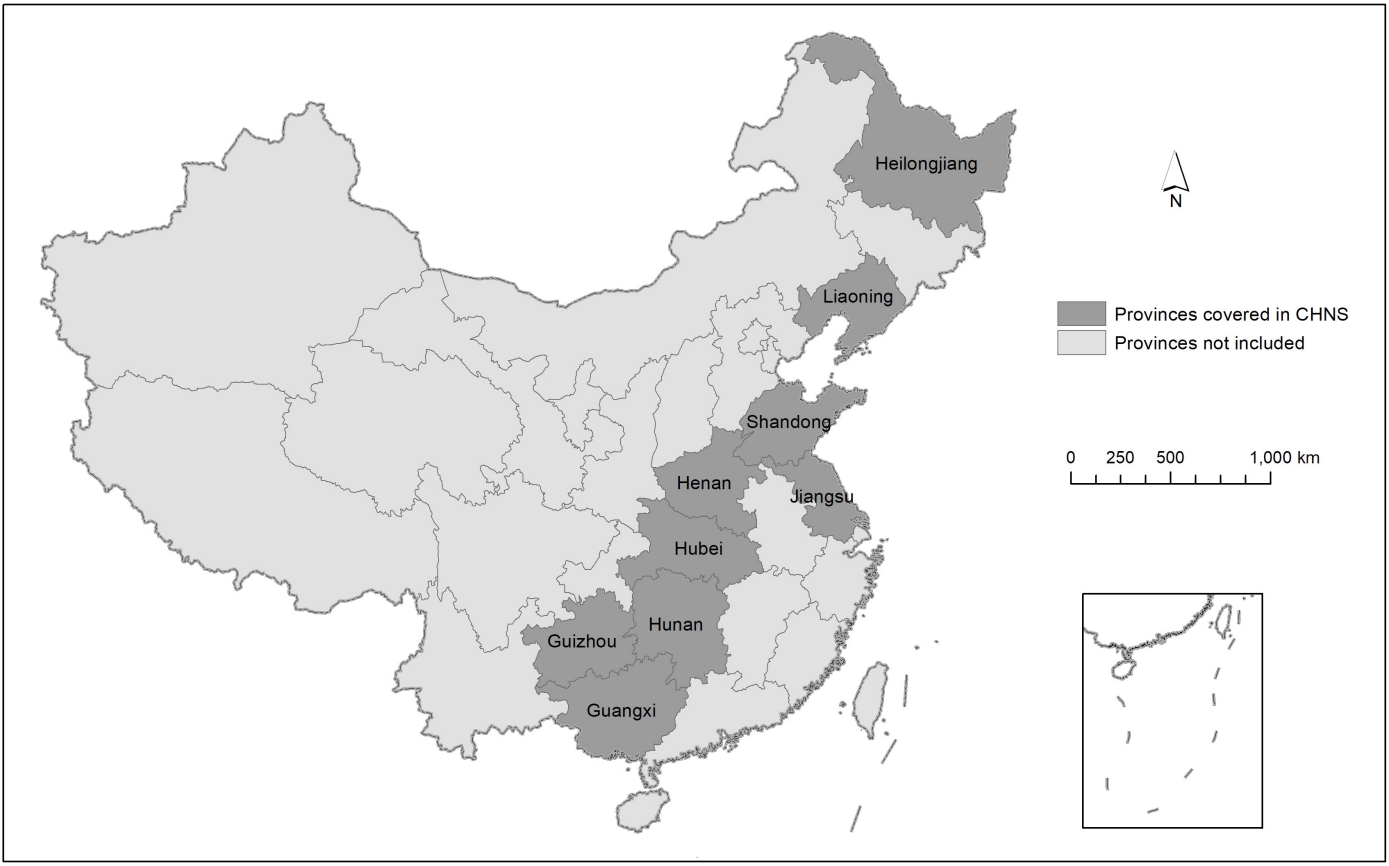
Provinces covered in the China Health and Nutrition Survey (CHNS).

An international team of researchers, specializing in nutrition, public health, economics, sociology, and/or demographics, conduct the survey, and each year, it is composed over 7 days using a multistage, random cluster process in provinces and cities that vary widely in geography, economic development, public resources, and health indicators. The smallest unit in the survey is the community, and each individual has a six-digit code representing their community: The first two digits indicate the province, the third classifies the rural or urban area, the fourth reflects the serial number of the rural or urban area in the region, and the last two digits represent the serial number of the individual's community in the province. Thus, we can distinguish whether the individuals in the province are in the same region or rural area, and we also know the specific name or location of the city or rural area. Overall, this article covers nine provinces across China: Guangxi Zhuang Autonomous Region, Henan, Hubei, Hunan, Guizhou, Heilongjiang, Shandong, Jiangsu, and Liaoning, among which the coastal provinces are Guangxi Zhuang Autonomous Region, Shandong, Jiangsu, and Liaoning (Figure 3). The provinces covered by CHNS accounted for 40.02% of the national GDP in 2001 and are widely distributed, so they are typical of other provinces. Additionally, the database contains individual illness/injury status, education level, gender, height, weight, and age. As this study considers adult illness/injury, we retain the 18–65 years age range sample in the baseline regressions and further test the 18–60 years sample in the robustness analysis.

This article also uses some regional-level indicators as control variables, including GDP per capita, the share of secondary industry in GDP, and environmental emission data, stemming from the China Statistical Yearbook. The Chinese Customs Databases from the General Administration of Customs of the People's Republic of China provide the provincial export data. Table 1 displays the variable definitions and data sources. Table 2 shows the statistical descriptions of the main variables.

**Table 1:** Definition of main variables and data sources.

**Variables**	**Definitions**	**Data sources**
**Individual level**		
Illness/injury	Dummy for illness or injury in the last 4 weeks	CHNS
LnAge	Log of age	CHNS
LnEducation	Log of years of education	CHNS
Gender	1 for men, 0 for women	CHNS
LnHeight	Log of height (cm)	CHNS
LnWeight	Log of weight (KG)	CHNS
Insurance	Do you have medical insurance? Yes 1, no 0	CHNS
**Household level**		
Tap water	Drinking water is obtained from the tap water to take 1, otherwise take 0	CHNS
Toilet condition	1 for household use of toilets indoors, 0 otherwise	CHNS
Light	1 for households with electric lights, 0 otherwise	CHNS
Income_pc	Log of per capita household income ($1,000)	CHNS
**Community level**		
Popu_ Index	Community Population Density Category	CHNS
Health_Index	Community health service quality	CHNS
Sani_Index	Sanitation Score	CHNS
**Regional level**		
SO_2_	SO_2_ emissions per capita	National Bureau of Statistics
Ind_Water	Industrial wastewater discharge per capita	National Bureau of Statistics
Export_Ratio	1 for the export/GDP value greater than the median, 0 otherwise	Chinese Customs Databases
Pergdp	GDP per capita	National Bureau of Statistics
Industrialization	Ratio of industrial value added to GDP	National Bureau of Statistics

**Table 2:** Summary statistics of the main variables.

**Variable**	**Mean**	**Sd**	**Min**	**Max**
**Panel A individual level**
Illness/injury	0.101	0.301	0	1
LnAge	3.704	0.318	2.890	4.174
LnEducation	1.972	0.783	0	3.135
Gender	0.483	0.500	0	1
LnHeight	5.080	0.0520	4.174	5.244
LnWeight	4.072	0.173	3.292	4.776
Insurance	0.313	0.464	0	1
**Panel B household level**
Tap water	0.705	0.456	0	1
Toilet condition	0.358	0.480	0	1
Light	0.987	0.115	0	1
Income_pc	8.043	1.044	0.223	12.32
**Panel C community level**
Popu_Index	5.853	1.438	0.500	10
Health_Index	5.395	2.305	0	10
Sani_Index	6.221	3.136	0.300	10
**Panel D regional level**
SO_2_	0.014	0.005	0.006	0.0280
Ind_Water	0.002	0.001	0	0.004
Export_Ratio	0.543	0.505	0	1
Pergdp	8.586	0.435	7.635	9.220
Industrialization	0.481	0.0750	0.365	0.664

## Results

### Baseline Results

The baseline results in this article demonstrate that export expansion may lead to increased illness/injury among Chinese adults. Table 3 shows the empirical results based on Equation (1). In column (1), where we do not include any control variables or fixed effects, the estimated coefficient of Export_Ratio^*^WTO is significantly positive, indicating that export expansion, stemming from trade liberalization, may lead to an increase in illness/injury. In columns (2)–(6), we add year and community fixed effects (column 2), as well as district-level (column 3), community-level (column 4), household-level (column 5), and individual-level control variables (average effect (AE): 1.83%; 95% CI: 0.38–3.28%) (column 6). We find that the variables' estimated coefficients are still significantly positive. Thus, our baseline results remain robust.

**Table 3:** Baseline results.

**Variable**	**(1)**	**(2)**	**(3)**	**(4)**	**(5)**	**(6)**
Export_Ratio^*^WTO	0.121^***^	0.138^***^	0.126^***^	0.118^***^	0.127^***^	0.113^**^
	(2.896)	(3.141)	(2.817)	(2.6214)	(2.802)	(2.466)
Export_Ratio	−0.010	−0.316	0.331	0.287	0.261	0.106
	(−0.278)	(−1.520)	(0.746)	(0.5972)	(0.538)	(0.214)
WTO	0.266^***^	0.214^***^	0.233^***^	0.249^***^	0.260^***^	0.182^***^
	(8.685)	(5.580)	(5.170)	(5.2275)	(5.366)	(3.660)
Pergdp			−0.525^*^	−0.511^*^	−0.486	−0.448
			(−1.712)	(−1.6594)	(−1.573)	(−1.423)
Industrialization			0.565	0.485	0.517	0.498
			(1.250)	(1.0666)	(1.135)	(1.077)
Popu_Index				0.006	0.007	0.011
				(0.1838)	(0.224)	(0.352)
Health_Index				0.012	0.011	0.013
				(1.5575)	(1.458)	(1.624)
Sani_Index				−0.003	0.001	−0.002
				(−0.2740)	(0.090)	(−0.149)
Tap water					−0.056	−0.055
					(−1.489)	(−1.428)
Toilet condition					−0.040	−0.019
					(−1.065)	(−0.491)
Light					−0.163^*^	−0.156^*^
					(−1.867)	(−1.760)
Income_pc					−0.009	−0.015
					(−0.726)	(−1.172)
LnAge						0.745^***^
						(15.256)
LnEducation						−0.023
						(−1.229)
Gender						−0.072^**^
						(−2.189)
LnHeight						−0.145
						(−0.392)
LnWeight						−0.105
						(−1.186)
Insurance						0.118^***^
						(4.124)
Constant	−1.466^***^	−1.244^***^	2.606	2.449	2.502	0.659
	(−59.171)	(−8.508)	(1.121)	(1.0412)	(1.060)	(0.220)
Year fixed effects	No	Yes	Yes	Yes	Yes	Yes
Community	No	Yes	Yes	Yes	Yes	Yes
fixed effects						
Observations	28,383	27,997	27,997	27,997	27,997	27,997
Pseudo R-squared	0.0151	0.0745	0.0746	0.0748	0.0752	0.101

*The z-statistics are, respectively, reported in parentheses below the estimated coefficients*.

***, **, and * indicate significance at 1, 5, and 10% levels, respectively.

### Robustness Checks

This study examines the impact of export expansion on the illness/injury of Chinese adults. However, the definition of the adult age range may have a different impact on the results because adults of different ages are affected by export expansion differently. The sample's age range selected in the baseline regression is 18–65 years, while the retirement age of adult Chinese men is 60 years old. Therefore, as seen in column (1) of Supplementary Table S1, this study selected a sample with an age range of 18–60 years old. The results are still robust, indicating that the different ways of defining the adult age range do not affect the results of this study.

Moreover, we use the probit model in the baseline regression to further test the effect of export expansion on adult illness/injury. To exclude the impact of model choice on the results, we further employ a logit model in column (2) of Supplementary Table S1 for robustness analysis. The results are consistent with the benchmark regression, indicating that model choice does not impact our findings.

Moreover, to avoid the effect of discrete variables, we examine the interaction between the continuous variable (export/GDP value by province) and WTO membership or no membership in column (3). We find that the variables are significantly positive for both sets of results, indicating that the findings remain robust to different ways of classifying experimental and control groups. Finally, we cluster the SEs to the individual level in the baseline regression, which can handle potential heteroskedasticity. However, because we only cluster to the individual level, we must ensure no serial correlation between individuals. We relax this assumption by clustering the SEs to the household level, making the serial correlation between individuals within households permissible (31, 35). Column (4) shows that the results and the main findings remain significantly positive.

#### A Parallel Trend Assumption

The most important assumption underlying causal identification in the DID model is a parallel trend assumption: ensuring that the same trend exists between experimental and control groups before the event. In the baseline regression, we use the interaction term constructed from the export shocks and the time dummy variable for WTO accession to identify the average changes in adult illness/injury in both the groups before and after WTO accession. To test the parallel trend hypothesis, we construct the following dynamic model.


(2) $$\begin{array}{l} \text{Illness}/{\text{injury}}_{ihcpt}\;=\;\alpha +\beta_{t}{\sum^{\text{​}}}^{\text{​}}{}_{Year=2000,2004,2006}Export\_Ratio_{p}\\ \;\times \;Year_{t}+\rho Export\_Ratio_{p}+X_{i}^{{}^{\prime }}\delta +X_{h}^{{}^{\prime }}\gamma \\ \begin{array}{cc} \;+ & X_{c}^{{}^{\prime }}\kappa +X_{p}^{{}^{\prime }}\eta +\lambda_{c}+\lambda_{t}+\varepsilon_{ihcpt} \end{array} \end{array}$$

Equation (2) replaces *Export*_*Ratio*_*p*_ × *WTO*_*t*_ in Equation (1) with the interactions between export ratio and the entire set of time dummy variables. The results in Supplementary Table S2 show that the estimated coefficients are insignificant before trade liberalization, significantly positive in 2004 after trade liberalization, and insignificant in 2006, suggesting that the parallel trend hypothesis is satisfied and that the differences in exposure to export shocks between provinces are diminishing. In other words, the regions that experienced minor export shocks at the beginning of trade liberalization were exposed to the same shocks in 2006 as the regions had previously higher exportation.

#### Control for Other Simultaneous Policies

In addition to the impact of export expansion, induced by China's WTO accession, the policy shocks occurring during the same period include the relaxation of restrictions on foreign investment and reform of state-owned enterprises in the early 2000's. Thus, we add two control variables (i.e., the logarithm of the number of foreign-invested enterprises and the ratio of the number of state-owned enterprises to the number of domestic enterprises) in columns (1) and (2) of Supplementary Table S3 (36, 37). Our results remain robust after adding these control variables. China's accession to WTO also affects the import volume of each region in China. Thus, the benchmark result may have also been affected by import shock. To consider the potential impact of import shock, we control the import amount of each region in China in column (3), and the estimation result shows that the variable is still significantly positive after controlling the import shock. When we add all of the above control variables in column (4), the results remain consistent with the baseline results, suggesting that export shocks may significantly increase adult illness/injury.

#### Placebo Test

For a placebo test, we mainly follow Topalova (31) to study whether export expansion would have an impact on adult illness/injury if China's entry into the WTO happened in 1998. To eliminate the effects of trade liberalization in 2001, we retained only the samples that had been collected prior to 2001 for the placebo test, which results in only half of the observations. The export shocks induced by China's accession to the WTO did not occur in 1998. Thus, we expect the variable of interest to be insignificant; otherwise, it could indicate that the other potential factors we have not identified may be contributing to the impact of export shocks on adult illness/injury. The result shows that export shocks do have an insignificant impact on adult illness/injury in the pre-WTO period in column (1) of Supplementary Table S4. To avoid the influence of the sample, we only use the data of the two periods before and after China's accession to the WTO, and the results remain robust in column (2). The estimates for columns (1) and (2) only retain the data from the two periods, but their sample sizes are still different due to the differences in the number of samples surveyed every year.

#### Two Periods Estimation

The DID model in baseline regression may overestimate the significance of the estimated coefficients because of sequential correlation problems. To overcome this problem, we follow Bertrand et al. (38) to adopt a two-period estimation model, which collapses the panel data into two periods, one before and the other after China's accession to the WTO. The variables in the two periods are averaged to construct a two-period DID sample. The estimated results in column (1) of Supplementary Table S5 are consistent with baseline regression.

#### Discussion Regarding the Effects of Exports

As exports affect income and/or GDP per capita, the inclusion of both items in Equation (1) is potentially misleading. To address this potential issue, we tried two approaches: (a) try Equation (1) without the income and GDP variables in column (1) of Supplementary Table S5, and (b) do a side calculation to see how export growth influences income and GDP per capita growth in columns (2) and (3). The interaction term in column (1) remains significantly positive, indicating that our findings are robust. Based on the coefficients estimated earlier, we can calculate that the total export effect is 0.125, indicating that the export expansion caused by trade liberalization had a positive impact on adult illness/injury.

### Discussion on Mechanisms

In this section, we explore the mechanisms through which export expansion impacts adult illness/injury. First, we examine whether pollutant emissions (e.g., industrial sulfur dioxide and industrial wastewater) are considered as a mechanism through which export expansion affects adult illness/injury. According to the results in columns (1) and (2) of Table 4, the interaction between *Export*_*ratio*_*p*_ × *WTO*_*t*_ and industrial sulfur dioxide emissions is significantly positive, suggesting that industrial sulfur dioxide emissions are considered as a mechanism through which export expansion affects adult illness/injury.

**Table 4:** Discussion on mechanisms.

**Variable**	**(1)**	**(2)**	**(3)**	**(4)**	**(5)**
Export_Ratio^*^WTO^*^SO2	21.902^**^				
	(2.116)				
Export_Ratio^*^SO2	−58.546^***^				
	(−5.398)				
WTO^*^SO2	6.697				
	(1.064)				
Export_Ratio^*^WTO^*^Ind_water		−79.898			
		(−0.902)			
Export_Ratio^*^Ind_water		−287.480^***^			
		(−3.057)			
WTO^*^Ind_water		180.215^**^			
		(2.499)			
Export_Ratio^*^WTO^*^Unemploy			0.012		
			(0.148)		
Export_Ratio^*^Unemploy			0.140^**^		
			(2.345)		
WTO^*^Unemploy			0.071		
			(1.463)		
Export_Ratio^*^WTO^*^Hour				0.008^*^	
				(1.858)	
Export_Ratio^*^WTO				−0.008^**^	
				(−2.051)	
WTO^*^Hour				−0.003	
				(−1.528)	
Export_Ratio^*^WTO^*^Wage					−0.000
					(−1.454)
Export_Ratio^*^Wage					0.000
					(0.677)
WTO^*^Wage					0.000
					(1.360)
Export_Ratio^*^WTO	−0.115	0.239	0.096^*^	−0.060	0.377^***^
	(−0.737)	(1.581)	(1.818)	(−0.301)	(2.953)
Control variables	Yes	Yes	Yes	Yes	Yes
Year fixed effects	Yes	Yes	Yes	Yes	Yes
Community fixed effects	Yes	Yes	Yes	Yes	Yes
Observations	27,997	27,997	27,908	11,720	6,023
Pseudo R-squared	0.103	0.102	0.103	0.106	0.0906

*The z-statistics are, respectively, reported in parentheses below the estimated coefficients*.

***, **, and * indicate significance at 1, 5, and 10% levels, respectively.

Second, we analyze whether the labor market (e.g., unemployment, working hours, and wages) is a mechanism. The results in column (3) reveal that the interaction between unemployment, export ratio, and the time of WTO accession dummy variable is not significant, indicating that adult employment is not a mechanism through which export expansion affects adult illness/injury. As the variable of interest in column (4) is significantly positive, it appears that working hours is a mechanism through which export expansion affects adult illness/injury (39–41). Finally, the variable of interest in column (5) is insignificantly negative, highlighting that adult wages are not a mechanism through which export expansion affects adult illness/injury. In summary, industrial sulfur dioxide emissions and labor hours are the mechanisms through which export expansion associated with WTO accession increases adult illness/injury.

### Discussion on Heterogeneity

In this section, we analyze the heterogeneity of the effects within different dimensions. To examine the heterogeneity of individuals in rural or urban contexts, we divide the data into two categories according to whether the individuals were in either area. The results from columns (1) and (2) of Table 5 show that export expansion did not significantly affect adult illness/injury in rural areas, but in the urban individuals' sample, it significantly increased the likelihood of adult illness/injury (AE: 5.32%; 95% CI: 2.46–8.18%).

**Table 5:** Discussion on heterogeneity.

**Variable**	**(1)**	**(2)**	**(3)**	**(4)**	**(5)**	**(6)**
	**Rural**	**Urban**	**Female**	**Male**	**Low-income**	**High-income**
Export_Ratio^*^WTO	0.016	0.307^***^	0.157^**^	0.064	0.011	0.332^***^
	(0.280)	(3.629)	(2.481)	(0.946)	(0.099)	(3.430)
Control variables	Yes	Yes	Yes	Yes	Yes	Yes
Year fixed effects	Yes	Yes	Yes	Yes	Yes	Yes
Community fixed effects	Yes	Yes	Yes	Yes	Yes	Yes
Observations	20,082	7,915	14,326	13,058	12,969	12,804
Pseudo R-squared	0.103	0.104	0.114	0.104	0.136	0.107

*The z-statistics are, respectively, reported in parentheses below the estimated coefficients*.

***, **, and * indicate significance at 1, 5, and 10% levels, respectively.

Columns (3) and (4) demonstrate that export expansion had significantly positive effects on female adult illness/injury (AE: 2.68%; 95% CI: 0.57–4.80%). We further examine the effects with the dimension of income, taking the median income as the measurement standard to distinguish between high- (value of 1) and low-income groups (value of 0). The results in columns (5) and (6) reveal that export expansion's effect on adult illness/injury is not only statistically significant in the low-income group, but also significantly positive in the high-income group (AE: 5.90%; 95% CI: 2.53–9.27%).

## Discussion

### Conclusion

As few studies systematically and comprehensively analyze the effect of export expansion, stemming from trade liberalization, on adult illness/injury, we use China's accession to the WTO as a quasi-natural experiment and employ the DID method to explore this impact. We also comprehensively discuss the impact mechanisms and heterogeneity of trade liberalization from multiple perspectives. Overall, we find that export expansion, induced by China's WTO accession, may have a significantly positive effect on adult illness/injury (AE: 1.83%; 95% CI: 0.38–3.28%), a finding that holds after a series of robustness tests. We further explore a few possible mechanisms, such as pollutant emissions, and working hours. The results reveal that pollutant emissions and working hours are the main mechanisms through which export expansion increases adult illness/injury. Finally, through a heterogeneity analysis, we find that urban residents (AE: 5.32%; 95% CI: 2.46–8.18%), women (AE: 2.68%; 95% CI: 0.57–4.80%), and high-income groups (AE: 5.90%; 95% CI: 2.53–9.27%) are more vulnerable to export shocks than their counterparts, making them more likely to suffer from diseases.

We find a statistically significant and positive effect of export expansion on adult illness/injury. In line with the findings of this study, Fan et al. (27) found that the reduction of import tariffs resulting from trade liberalization adversely affected workers' health, demonstrating a negative impact of trade on adult health from the perspective of import tariffs. Bombardini and Li (29) discovered that trade-induced export pollution shocks significantly increased infant and child mortality, suggesting that the adverse health effects of trade may extend not only to adults, but also to infants and children.

Export expansion significantly increased the likelihood of adult illness/injury in the urban individuals' sample. Two reasons can explain this result: (1) pollution stemming from export expansion has less of an effect in rural areas, and/or (2) urban residents are more likely to work in industries related to export trade, which leads to longer working hours. Export expansion had significantly positive effects on female adult illness/injury. The possible reasons for this are that women's work intensity increases more under export shocks. We find that export expansion's effect on adult illness/injury is not statistically significant in the low-income group, but significantly positive in the high-income group. One possible reason for this result is the high-income group's longer working hours under the impact of export expansion, leading to increases in illness/injury.

### Limitations

This article concludes with the following limitations. First, we use the DID method to identify the relative but not the absolute effects of the export shocks resulting from trade liberalization on adult illness/injury. Second, these conclusions are particularly relevant to developing countries, including China, and may not be generalizable to other countries. During the early stages of economic development, China did not coordinate economic development with environmental protection, which caused export shocks to negatively affect adult health. As long as countries like China balance environmental protection and health with economic development, it is likely that the expansion of exports will not adversely impact adult health. Third, there may be potential threats not considered in this study that lead to the bias of the estimated coefficients. We alleviate the endogenous problem using the DID method. Moreover, to ensure the validity of causal inference, we have conducted many robustness checks, including the control for other simultaneous policies and the placebo test. Fourth, the issue of measuring export shocks using a binary indicator of export/GDP relative to the median will result in our findings identifying only the relative effects of experimental and control groups based on this indicator.

### Policy Recommendations

Our conclusions have important policy implications. Economic growth is vital for any country, whether it is developing or developed. Although China's economy has achieved great success after the reform and opening-up process, especially after joining the WTO, the high speed of economic development has been implemented at the expense of the environment. Our findings can encourage policymakers to develop better policies that balance economic development, environmental protection, and adult illness/injury in developing countries.

First, during the early stages of economic development, many countries tend to place more emphasis on the speed of economic development than on environmental protection, which can cause severe environmental damage and health risks to residents. Our findings demonstrate that China's export trade has deteriorated the ecological environment and increased the likelihood of adult diseases. Therefore, developing countries, particularly China, should shift from extensive to intensive economic development, and build their economies while simultaneously coordinating environmental protection and paying attention to the health of their residents. In addition, governments should invest more in health in trade negotiations and full health impact assessments, and undertake an array of governance reforms to ensure that health is adequately considered in cost-benefit analyses (42–45). In this way, China will achieve sustainable economic development and improve the health of its citizens.

Second, despite the fact that the health status of Chinese people is generally improving, there are still many factors that negatively affect their health. This study finds that pollutant emissions and employee working hours are the mechanisms through which export expansion, induced by trade liberalization, increases adult illness/injury. Therefore, we suggest that stronger environmental regulation and better job security should accompany economic development and policies should be formulated in full consideration of their impact on different regions. If more stringent environmental regulations were implemented in developed areas, these areas would attempt to evade the environmental regulations through industrial transfer, which will cause further damage to the environment in less developed areas. Moreover, technological innovation is an important tool for balancing economic development with environmental protection.

## Data Availability Statement

The China Health and Nutrition Survey data is available at the Carolina Population Center at the University of North Carolina at Chapel Hill (https://www.cpc.unc.edu/projects/china/data/datasets/index.html), and the China Statistical Yearbook is available at the China National Knowledge Infrastructure (CNKI) platform (https://data.cnki.net/Trade/yearbook/single/). While the raw data of the Chinese Customs Database used in this study are available on request from the corresponding author, and are not publicly available due to privacy.

## Author Contributions

HC and JX conceptualized the study and contributed to methodology. JL contributed to supervision and funding acquisition. JX contributed to software. HC contributed to formal analysis and writing—original draft preparation. HC and JL contributed to writing, reviewing, and editing. All authors contributed to the article and approved the submitted version.

## Funding

This research was supported by the Social Science Research Base Program of Fujian at the Research Center of Public Service Quality of Xiamen University (Grant No. FJ2020JDZ006) and the National Natural Science Foundation of China (Grant No. 42101199).

## Conflict of Interest

The authors declare that the research was conducted in the absence of any commercial or financial relationships that could be construed as a potential conflict of interest.

## Publisher's Note

All claims expressed in this article are solely those of the authors and do not necessarily represent those of their affiliated organizations, or those of the publisher, the editors and the reviewers. Any product that may be evaluated in this article, or claim that may be made by its manufacturer, is not guaranteed or endorsed by the publisher.
