# Supplementary material for: Export Expansion May Increase Adult Illness and Injury: A Quasi-Natural Experiment on China's Accession to the World Trade Organization
Source: Front Public Health. 2022 Apr 5;10:798686. doi: 10.3389/fpubh.2022.798686 (PMC9016144; doi:10.3389/fpubh.2022.798686)
Supplement: Supplementary file 1 [file Data_Sheet_1.docx]

Supplementary Material

**Table S1 |** Robustness checks.

| **Variable** | **(1)** | **(2)** | **(3)** | **(4)** |
| --- | --- | --- | --- | --- |
| Export_Ratio*WTO | 0.123** | 0.235*** |  | 0.113** |
|  | (2.506) | (2.587) |  | (2.250) |
| Export_Ratio_Con*WTO |  |  | 1.964*** |  |
|  |  |  | (4.342) |  |
| Redefining the range of adults | YES |  |  |  |
| Logit Model |  | YES |  |  |
| Continuous export value |  |  | YES |  |
| Standard errors clustered at the household level |  |  |  | YES |
| Control variables | Yes | Yes | Yes | Yes |
| Year fixed effects | Yes | Yes | Yes | Yes |
| Community fixed effects | Yes | Yes | Yes | Yes |
| Observations | 25667 | 27997 | 27997 | 27997 |
| Pseudo R-squared | 0.0970 | 0.101 | 0.102 | 0.101 |

The z-statistics are respectively reported in the parentheses below the estimated coefficients. ***, **, and * indicate significance at the 1%, 5%, and 10% levels, respectively.

**Table S2 |** The parallel trend assumption.

| **Variable** | **(1)** |
| --- | --- |
| Export_Ratio * Year 2000 | -0.014 |
|  | (-0.213) |
| Export_Ratio * Year 2004 | 0.216*** |
|  | (3.440) |
| Export_Ratio * Year 2006 | -0.031 |
|  | (-0.466) |
| Export_Ratio | 0.198 |
|  | (0.401) |
| Control variables | Yes |
| Year fixed effects | Yes |
| Community fixed effects | Yes |
| Observations | 27997 |
| Pseudo R-squared | 0.102 |

The z-statistics are respectively reported in the parentheses below the estimated coefficients. ***, **, and * indicate significance at the 1%, 5%, and 10% levels, respectively.

**Table S3 |** Control for other simultaneous policies.

| **Variable** | **(1)** | **(2)** | **(3)** | **(4)** |
| --- | --- | --- | --- | --- |
| Export_Ratio*WTO | 0.169** | 0.203*** | 0.205*** | 0.207*** |
|  | (2.514) | (3.076) | (2.855) | (2.773) |
| FDI(log) | -0.055 |  |  | -0.052 |
|  | (-0.936) |  |  | (-0.841) |
| SOE share |  | -1.000*** |  | -1.171*** |
|  |  | (-3.153) |  | (-2.764) |
| Import |  |  | -0.000* | 0.000 |
|  |  |  | (-1.837) | (0.692) |
| Control variables | Yes | Yes | Yes | Yes |
| Year fixed effects | Yes | Yes | Yes | Yes |
| Community fixed effects | Yes | Yes | Yes | Yes |
| Observations | 19195 | 19195 | 19195 | 19195 |
| Pseudo R-squared | 0.103 | 0.103 | 0.103 | 0.104 |

The z-statistics are respectively reported in the parentheses below the estimated coefficients. ***, **, and * indicate significance at the 1%, 5%, and 10% levels, respectively.

**Table S4 |** Placebo test.

| **Variable** | **(1)** | **(2)** |
| --- | --- | --- |
| Export_Ratio * Year 1998 | 0.023 |  |
|  | (0.305) |  |
| Export_Ratio*WTO |  | 0.252*** |
|  |  | (3.902) |
| Control variables | Yes | Yes |
| Year fixed effects | Yes | Yes |
| Community fixed effects | Yes | Yes |
| Observations | 11785 | 13544 |
| Pseudo R-squared | 0.122 | 0.125 |

The z-statistics are respectively reported in the parentheses below the estimated coefficients. ***, **, and * indicate significance at the 1%, 5%, and 10% levels, respectively.

**Table S5 |** Two periods estimation

| **Variable** | **(1)** |
| --- | --- |
| Export_Ratio*WTO | 0.111** |
|  | (1.960) |
| Control variables | Yes |
| Year fixed effects | Yes |
| Community fixed effects | Yes |
| Observations | 17889 |
| Pseudo R-squared | 0.107 |

The z-statistics are respectively reported in the parentheses below the estimated coefficients. ***, **, and * indicate significance at the 1%, 5%, and 10% levels, respectively.

**Table S5 |** Discussion regarding the effects of exports

| **Variable** | **(1)** | **(2)** | **(3)** |
| --- | --- | --- | --- |
|  | Illness/ injury | Pergdp | Income_pc |
| Export_Ratio*WTO | 0.128*** | -0.024*** | -0.086*** |
|  | (2.814) | (-17.979) | (-3.951) |
| Export_Ratio | -0.500* | 1.328*** | 0.796*** |
|  | (-1.821) | (245.054) | (6.027) |
| WTO | 0.150*** | 0.052*** | 0.423*** |
|  | (3.260) | (42.659) | (19.141) |
| Control variables | Yes | Yes | Yes |
| Year fixed effects | Yes | Yes | Yes |
| Community fixed effects | Yes | Yes | Yes |
| Observations | 27997 | 28383 | 28383 |
| (Pseudo) R-squared | 0.101 | 0.994 | 0.340 |
